# National Norms for Hospital Frailty Risk Score Among Hospitalized Adults in the USA

**DOI:** 10.1007/s11606-025-09483-w

**Published:** 2025-03-31

**Authors:** Christine Loyd, Taylor Miller, Shrest Nath, Yue Zhang, Richard E. Kennedy

**Affiliations:** 1 Department of Clinical and Diagnostic Sciences, University of Alabama at Birmingham, Birmingham, AL USA; 2 Department of Medicine, Division of Gerontology, Geriatrics, and Palliative Care, University of Alabama at Birmingham, Birmingham, AL USA

**Keywords:** Hospital Frailty Risk Score (HFRS), National norms, Adult inpatients, Frailty assessment

## Abstract

**Background:**

Frailty among inpatients increases risk for hospital-associated disability and death. Yet, frailty is not regularly screened in acute care due to the lack of standardized methods, the complexity of frailty, and time and energy required of hospital personnel. Thus, screening with routinely collected data provides an opportunity to assess frailty across inpatient populations.

**Objective:**

To calculate normative values for Hospital Frailty Risk Score (HFRS) among adult inpatients in the USA based on age, sex, and race.

**Design:**

A retrospective cross-sectional analysis of the 2018 National Inpatient Sample (NIS) database.

**Patients:**

US adult inpatients aged 18y + with a focus on patients aged at least 45.

**Main Measures:**

Hospital Frailty Risk Score (HFRS) is a validated measure that uses ICD-10 codes to calculate frailty risk among hospitalized patients.

**Key Results:**

Mean HFRS significantly increased with increasing age across sex and race (*p* < 0.001). Among the oldest age groups 65y + , mean and median normative values were similar between male and female inpatients (mean HFRS range, 6.71–9.62; median HFRS range, 5.40–8.70), and Black inpatients had the highest frailty risk compared to other races (mean HFRS range = 7.56–10.47; median HFRS range = 6.30–9.50). Asian/Pacific Islander inpatients had similar frailty risk to Black inpatients among those 90y + (mean HFRS = 10.48; median HFRS = 9.50).

**Conclusions:**

The US national norms for HFRS provide a standardized reference tool for comparing frailty risk among clinical and research inpatient populations to a typical hospitalized adult for their age, sex, and race.

## INTRODUCTION

Frailty is a state of cumulative decline in physiologic reserve capacity that results in diminished ability to restore homeostasis following a stressor event.^1,2^ Abundant evidence supports that being frail increases risk for a variety of negative health outcomes for patients,^3–8^ and predicts adverse outcomes among hospitalized and postoperative patients.^9,10^ The prevalence of frailty increases with age and is most commonly observed among older adults (65y +).^11^ However, evidence also supports that younger adults with multimorbidity (having at least two chronic health conditions) are at risk for frailty.^12^ Other risk factors for frailty include female sex, socioeconomic status, and number of health issues.^13,14^

Assessment of frailty in the acute care setting is of particular concern because many hospitalized patients are older and have multimorbidity.^15^ Furthermore, frailty increases risk for longer hospitalizations, development of hospital-associated disability, and death while in the hospital or after discharge.^10,16^ Identifying frailty among hospitalized patients can assist in targeting therapeutic resources to patients that would benefit most, which has the potential to benefit both the patient and the healthcare system. Despite this, frailty is not routinely screened when patients are hospitalized in acute care.^17^ Key barriers to frailty screening are lack of a gold standard assessment,^18^ the complex nature of frailty which includes psychosocial and physical components,^19^ and the fact that time and energy among hospital personnel are limited in the acute care setting so that screening for frailty might not be prioritized.^20^

Most frailty assessment methods are complicated and time-intensive, and require training of hospital personnel to ensure interrater reliability.^21–23^ Use of hospital administrative data is a potential method to circumvent these issues. The Hospital Frailty Risk Score (HFRS) was recently developed and validated to identify patients with frailty using diagnostic (ICD-10) codes, a universal coding system for medical conditions.^24^ ICD codes are routine data collected when patients are hospitalized, which allows frailty assessment without additional data collection and allows frailty analysis on a large scale. The objective of this investigation was to calculate the national norms for HFRS among adult inpatients using a US national sample stratified by age, sex and race to promote more widespread use of this instrument.

## METHODS

### Data Source

The Healthcare Cost and Utilization Project (HCUP) by the Agency of Healthcare Research and Quality (AHRQ) has resulted in development of the Nationwide Inpatient Sample (NIS) databases. The NIS is the largest publicly available all-payer inpatient dataset containing data from hospitals across the USA on more than 7 million hospitalizations each year.^25^ When weighted to the US population, the NIS dataset is representative of 97% of hospital discharges in the USA annually. The NIS includes data on inpatients admitted to acute care excluding observation stays. Admitted patients come from the community, from long-term and subacute care, and from rehabilitation facilities and reports on demographics, healthcare utilization, and diagnoses and procedures. Primary and secondary diagnoses are provided using International Classification of Diseases–10th Revision (ICD10) codes.^26^ The NIS database does not track readmissions of individual patients, so that each hospitalization is documented as being unique. The NIS dataset is provided in aggregate across hospitals and hospital admissions so this is not considered human subjects research by the UAB Institutional Review Board and thus not requiring protocol approval. Human ethics and consent to participate declarations: not applicable.

### Study Population

The study population were US adult patients hospitalized in 2018 at a hospital participating in providing data to the NIS. Demographic information (age, race, sex) and primary and secondary diagnoses via ICD-10 codes were extracted from the 2018 database and used in analysis. Since frailty is most common in older inpatients,^15^ this analysis focused on patients 45y and older by stratifying these patients into groups using 5-year intervals (45–49 years to 85–89 years). Patients aged 90y + were grouped into a single group as were patients aged 18–44 years. Patients were also grouped by sex (male, female) and race (White, Black, Hispanic, Asian or Pacific Islander, or Native American). The social construct of race was included in our analysis in light of evidence supporting that racial minority groups are at increased risk for poorer health outcomes.^27,28^

### Frailty Assessment

The Hospital Frailty Risk Score (HFRS) is a validated assessment for screening for frailty and calculating frailty risk among hospitalized patients.^24^ The HFRS is calculated using ICD-10 codes for 109 diagnoses and factors influencing health status (Z codes) identified as being associated with frailty risk and each ICD-10 code was assigned a value ranging from 0.1 to 7.1 relative to its relationship to frailty severity. The sum of the values for each diagnosis yield the HFRS, with higher summary scores corresponding to higher risk for frailty.^24^ The initial validation of the HFRS categorized scores into frailty risk groupings—low risk (< 5), intermediate risk (5–15), and high risk (˃15),^24^ however for this study, we have provided HFRS as a continuous variable to provide normative values. Previous validation studies have demonstrated higher scores on the HFRS are associated with longer hospital stays and greater 30-day readmissions and mortality.^24^

### Statistical Analysis

Characteristics of the study population were described using means and standard deviations (SD) for age as a continuous variable and frequencies (percentages) for categorical variables (age group, sex group, and race group). Differences between groups were analyzed using independent samples *t* tests for continuous variables and Pearson’s chi-square tests for categorical variables. Norms for HFRS were calculated as weighted means, SDs, and percentile points using the *survey* package version 4.2.0 in the R programming environment (R Core Development Team, Vienna, Austria).^29^ Weighting was performed using values supplied in the NIS dataset to provide norms for the US national inpatient population.^30^ Norms were stratified by 5-year age intervals to compare frailty across age groups, as well as sex and race.

## RESULTS

The characteristics of the study population (*N* = 6,051,974) are presented in Table 1. The mean age of the population was 58.2 years (SD = 27.3), and most inpatients were 45 years and older (72.1%) with the largest age groups being between age 55 and 79 years. The majority of inpatients were female (57.4%) and White (69%). The mean Hospital Frailty Risk Score (HFRS) for all inpatients was 6.55 (SD = 5.34).

**Table 1:** Study Population Characteristics Are Presented Including Mean Age (SD), Number of Hospitalizations (%) Based on Age Group, Race Group, and Sex Group, and Mean HFRS (SD). *HFRS* Hospital Frailty Risk Score

	All	*N*
	*N* = *6,051,974*	
Age (years), mean (SD)	58.2 (20.2)	6,051,974
Age group, no. (%)		6,051,974
18–44	1,687,772 (27.9%)	
45–49	293,937 (4.86%)	
50–54	378,534 (6.25%)	
55–59	492,857 (8.14%)	
60–64	551,201 (9.11%)	
65–69	578,068 (9.55%)	
70–74	568,088 (9.39%)	
75–79	505,829 (8.36%)	
80–84	420,564 (6.95%)	
85–89	326,616 (5.40%)	
90 and above	248,508 (4.11%)	
Sex, no. (%)		6,051,456
Male	2,580,153 (42.6%)	
Female	3,471,303 (57.4%)	
Race, no. (%)		5,721,912
White	3,949,442 (69.0%)	
Black	890,717 (15.6%)	
Hispanic	678,971 (11.9%)	
Asian or Pacific Islander	164,466 (2.87%)	
Native American	38,316 (0.67%)	
HFRS, mean (SD)	6.55 (5.34)	4,928,029

Tables 2, 3, and 4 illustrate mean HFRS across the study sample based on age, sex, and race. Table 2 shows that mean HFRS increased incrementally with increasing age (*p* < 0.001). The lowest mean score was among inpatients 18–44 years (4.03, SD = 4.07) and those 45–49 years age group (5.35, SD = 4.59) while the highest mean scores were among oldest inpatients 90y + (9.62, SD = 5.76). Table 3 shows that mean HFRS were higher among the male inpatients (6.69, SD = 5.33) compared to female (6.43, SD = 5.36; *p* < 0.001), and Table 4 demonstrates that the mean HFRS differed based on race and was lowest among Hispanic inpatients (6.10, SD = 5.22) and highest among Asian/Pacific Islander (6.74, SD = 5.75) and White inpatients (6.64, SD = 5.31; *p* < 0.001).

**Table 2:** Hospital Frailty Risk Score Based on Age Group. Characteristics of Age Groups Are Presented Including Mean Age (SD), Number of hospitalizations (%) based on sex and race group, and mean HFRS (SD). *HFRS* Hospital Frailty Risk Score

	**18–44**	**45–49**	**50–54**	**55–59**	**60–64**	**65–69**	**70–74**	**75–79**	**80–84**	**85–89**	**90 + **	***N***
	***N* = *2,741,296***	***N* = *293,937***	***N* = *378,534***	***N* = *492,857***	***N* = *551,201***	***N* = *578,068***	***N* = *568,088***	***N* = *505,829***	***N* = *420,564***	***N* = *326,616***	***N* = *248,508***	
Age (years), mean (SD)	31.1 (6.98)	47.1 (1.34)	52.1 (1.35)	57.1 (1.35)	62.0 (1.35)	67.0 (1.36)	71.9 (1.37)	76.9 (1.37)	81.9 (1.36)	86.9 (1.35)	90.0 (0.00)	6,051,974
Sex, no. (%):												6,051,456
Male	457,076 (27.1%)	146,691 (49.9%)	198,488 (52.4%)	264,458 (53.7%)	292,014 (53.0%)	295,731 (51.2%)	278,956 (49.1%)	240,169 (47.5%)	188,295 (44.8%)	133,848 (41.0%)	84,427 (34.0%)	
Female	1,230,406 (72.9%)	147,214 (50.1%)	180,009 (47.6%)	228,355 (46.3%)	259,153 (47.0%)	282,312 (48.8%)	289,112 (50.9%)	265,649 (52.5%)	232,259 (55.2%)	192,762 (59.0%)	164,072 (66.0%)	
Race, no. (%):												5,721,912
White	878,963 (56.0%)	169,865 (61.2%)	230,212 (64.2%)	316,824 (67.7%)	368,838 (70.4%)	407,235 (74.1%)	421,974 (77.9%)	382,573 (79.2%)	320,209 (79.7%)	255,149 (81.7%)	197,600 (83.1%)	
Black	310,757 (19.8%)	58,146 (20.9%)	73,080 (20.4%)	88,393 (18.9%)	90,586 (17.3%)	79,200 (14.4%)	61,718 (11.4%)	49,110 (10.2%)	37,585 (9.36%)	24,581 (7.87%)	17,561 (7.38%)	
Hispanic	297,819 (19.0%)	40,255 (14.5%)	44,720 (12.5%)	49,777 (10.6%)	49,624 (9.48%)	47,061 (8.56%)	42,769 (7.90%)	37,092 (7.68%)	31,335 (7.80%)	22,993 (7.36%)	15,526 (6.53%)	
Asian or Pacific Islander	66,449 (4.24%)	6867 (2.47%)	7730 (2.15%)	9221 (1.97%)	11,047 (2.11%)	12,882 (2.34%)	12,261 (2.26%)	11,817 (2.45%)	10,911 (2.72%)	8698 (2.79%)	6583 (2.77%)	
Native American	14,317 (0.91%)	2621 (0.94%)	3073 (0.86%)	3681 (0.79%)	3517 (0.67%)	3291 (0.60%)	2726 (0.50%)	2160 (0.45%)	1512 (0.38%)	888 (0.28%)	530 (0.22%)	
HFRS, mean (SD)	4.03 (4.07)	5.35 (4.59)	5.71 (4.79)	6.06 (4.93)	6.43 (5.13)	6.74 (5.26)	7.15 (5.37)	7.73 (5.54)	8.40 (5.68)	9.06 (5.77)	9.62 (5.76)	4,928,029

**Table 3:** Hospital Frailty Risk Score Based on Sex. Characteristics of Sex Groups Are Presented Including Mean Age (SD), Number of Hospitalizations (%) Based on Sex and Race Group, and Mean HFRS (SD). *HFRS* Hospital Frailty Risk Score

	Male	Female	*N*
	*N* = *2,580,153*	*N* = *3,471,303*	
Age (years), mean (SD)	61.4 (17.5)	55.8 (21.7)	6,051,456
Race, no. (%)			5,721,470
White	1,736,944 (71.1%)	2,212,196 (67.5%)	
Black	368,589 (15.1%)	522,053 (15.9%)	
Hispanic	263,069 (10.8%)	415,853 (12.7%)	
Asian or Pacific Islander	58,122 (2.38%)	106,329 (3.24%)	
Native American	15,938 (0.65%)	22,377 (0.68%)	
HFRS, mean (SD)	6.69 (5.33)	6.43 (5.36)	4,927,688

**Table 4:** Hospital Frailty Risk Score Based on Race. Characteristics of Race Groups Are Presented Including Mean Age (SD), Number of Hospitalizations (%) Based on Sex and Race Group, and Mean HFRS (SD). *HFRS* Hospital Frailty Risk Score

	White	Black	Hispanic	Asian or Pacific Islander	Native American	*N*
	*N* = *3,949,442*	*N* = *890,717*	*N* = *678,971*	*N* = *164,466*	*N* = *38,316*	
Age (years), mean (SD)	61.2 (19.6)	52.9 (19.2)	50.4 (20.6)	54.4 (21.4)	51.9 (19.0)	5,721,912
Sex, no. (%)						5,721,470
Male	1,736,944 (44.0%)	368,589 (41.4%)	263,069 (38.7%)	58,122 (35.3%)	15,938 (41.6%)	
Female	2,212,196 (56.0%)	522,053 (58.6%)	415,853 (61.3%)	106,329 (64.7%)	22,377 (58.4%)	
HFRS, mean (SD)	6.64 (5.31)	6.54 (5.48)	6.10 (5.22)	6.74 (5.75)	6.43 (5.03)	4,677,566

Tables 5 and 6 provide the normative values (mean, SD, 5th–95th percentile) for HFRS based on age group and sex or race group. Table 5 illustrates that mean HFRS and percentile values increased with age for males and females. For male inpatients, the mean range was 4.85 (SD = 4.44) among those 18–44 years to 9.60 (SD = 5.80) among those 90y + . The median range for males was 3.50 (18–44 years) to 8.70 (90y +). For female inpatients, the mean range was 3.49 (SD = 3.71) among those 18–44 years to 9.62 (SD = 5.74) among those 90y + . The median range for females was 2.10 (18–44 years) to 8.70 (90y +). The mean HFRS and the percentile values were marginally higher among male inpatients in the younger age groups (< 70 years) compared to females. However, among inpatients 70y + , the mean HFRS and percentiles were slightly higher among females.

**Table 5:** Normative HFRS by Sex Across Age Groups. Mean, SD, and Percentiles for Mean HFRS Are Presented. *HFRS* Hospital Frailty Risk Score

		Age groups										
Sex	Statistic	18–44	45–49	50–54	55–59	60–64	65–69	70–74	75–79	80–84	85–89	90 +
Male	Mean	4.85	5.49	5.78	6.10	6.47	6.76	7.14	7.70	8.34	9.02	9.60
	SD	4.44	4.69	4.83	4.98	5.18	5.29	5.37	5.54	5.67	5.78	5.80
	5th percentile	0.50	0.70	0.70	0.70	0.80	1.10	1.30	1.40	1.50	1.50	1.90
	25th percentile	1.70	2.00	2.20	2.30	2.50	2.70	3.00	3.40	4.00	4.60	5.20
	50th percentile (median)	3.50	4.10	4.40	4.70	5.10	5.40	5.90	6.50	7.20	8.00	8.70
	75th percentile	6.60	7.60	8.00	8.50	9.10	9.40	10.00	10.70	11.50	12.30	13.00
	95th percentile	13.80	14.80	15.40	15.90	16.70	17.10	17.60	18.50	19.20	20.00	20.50
Female	Mean	3.49	5.20	5.63	6.01	6.39	6.71	7.16	7.75	8.44	9.09	9.62
	SD	3.71	4.49	4.74	4.88	5.07	5.22	5.38	5.54	5.69	5.77	5.74
	5th percentile	0.40	0.50	0.50	0.60	0.70	0.90	1.10	1.40	1.50	1.50	1.90
	25th percentile	1.10	1.90	2.10	2.30	2.50	2.70	3.00	3.50	4.00	4.70	5.30
	50th percentile (median)	2.10	3.90	4.30	4.70	5.10	5.40	5.90	6.50	7.40	8.10	8.70
	75th percentile	4.60	7.20	7.80	8.40	8.90	9.40	10.00	10.80	11.70	12.40	13.00
	95th percentile	11.10	14.10	15.10	15.60	16.30	17.00	17.60	18.40	19.30	20.00	20.40

**Table 6:** Normative HFRS by Race Across Age Groups. Mean, SD, and Percentiles for Mean HFRS Are Presented. *HFRS* Hospital Frailty Risk Score

		Age groups										
Race	Statistic	< 45	45–49	50–54	55–59	60–64	65–69	70–74	75–79	80–84	85–89	90 +
White	Mean	4.00	5.23	5.59	5.93	6.28	6.55	6.99	7.58	8.27	8.95	9.50
	SD	4.00	4.52	4.71	4.86	5.04	5.14	5.28	5.44	5.59	5.68	5.67
	5th percentile	0.40	0.50	0.50	0.70	0.70	0.90	1.20	1.40	1.50	1.50	1.90
	25th percentile	1.50	1.90	2.00	2.30	2.30	2.60	2.90	3.30	3.90	4.60	5.20
	50th percentile (median)	2.60	3.90	4.20	4.60	4.90	5.20	5.70	6.40	7.20	8.00	8.60
	75th percentile	5.40	7.20	7.80	8.30	8.80	9.20	9.80	10.60	11.40	12.20	12.80
	95th percentile	12.20	14.30	15.00	15.60	16.20	16.70	17.30	18.10	18.90	19.70	20.10
Black	Mean	4.21	5.65	6.01	6.44	7.00	7.56	8.07	8.63	9.27	9.94	10.47
	SD	4.16	4.75	4.94	5.13	5.39	5.62	5.81	6.00	6.13	6.25	6.22
	5th percentile	0.40	0.50	0.70	0.70	1.00	1.30	1.40	1.50	1.50	1.80	2.20
	25th percentile	1.50	2.20	2.30	2.60	3.00	3.30	3.70	4.10	4.60	5.20	5.80
	50th percentile (median)	2.80	4.40	4.70	5.20	5.70	6.30	6.80	7.40	8.00	8.80	9.50
	75th percentile	5.80	7.80	8.30	8.90	9.60	10.40	11.10	11.80	12.70	13.60	14.20
	95th percentile	12.60	14.90	15.70	16.40	17.50	18.50	19.40	20.30	21.10	21.90	22.20
Hispanic	Mean	4.00	5.39	5.75	6.10	6.49	6.83	7.27	7.86	8.46	9.11	9.66
	SD	4.11	4.53	4.78	4.91	5.11	5.24	5.43	5.61	5.71	5.88	5.88
	5th percentile	0.40	0.50	0.50	0.70	0.80	0.90	1.20	1.40	1.50	1.50	1.90
	25th percentile	1.30	2.00	2.20	2.30	2.60	2.80	3.10	3.50	4.00	4.60	5.20
	50th percentile (median)	2.50	4.10	4.50	4.80	5.20	5.60	6.00	6.70	7.30	8.10	8.70
	75th percentile	5.50	7.50	8.00	8.50	9.10	9.50	10.10	10.90	11.70	12.50	13.10
	95th percentile	12.30	14.40	15.10	15.80	16.40	17.10	17.80	18.70	19.60	20.40	20.70
Asian or Pacific Islander	Mean	3.59	5.45	5.84	6.28	6.53	7.01	7.27	8.14	8.80	9.85	10.48
	SD	4.02	4.85	5.12	5.35	5.34	5.61	5.49	5.98	5.99	6.26	6.20
	5th percentile	0.40	0.50	0.50	0.70	0.80	1.00	1.20	1.40	1.40	1.80	2.20
	25th percentile	1.00	1.80	2.10	2.30	2.40	2.80	3.00	3.70	4.20	5.10	5.80
	50th percentile (median)	2.00	4.00	4.40	4.90	5.10	5.60	5.90	6.80	7.60	8.60	9.50
	75th percentile	4.80	7.60	8.10	8.70	9.10	9.70	10.10	11.20	12.10	13.40	14.10
	95th percentile	11.90	15.00	15.60	16.50	17.00	17.90	18.10	19.70	20.30	21.80	22.20
Native American	Mean	4.82	6.13	6.64	6.65	6.68	7.12	7.39	7.84	8.57	8.60	9.46
	SD	4.39	4.62	4.91	4.76	4.88	5.29	5.28	5.28	5.57	5.34	5.79
	5th percentile	0.40	0.70	0.70	0.90	1.00	1.10	1.40	1.40	1.50	1.50	1.50
	25th percentile	1.50	2.60	2.90	3.00	3.00	3.00	3.30	3.80	4.30	4.70	5.10
	50th percentile (median)	3.40	5.10	5.60	5.60	5.60	6.00	6.30	6.80	7.30	7.70	8.30
	75th percentile	6.90	8.40	9.30	9.40	9.50	9.90	10.30	10.80	12.10	11.70	13.40
	95th percentile	13.70	15.00	15.90	15.70	15.90	17.30	17.40	17.60	19.00	18.80	20.90

Table 6 demonstrates that mean HFRS and percentile values increase with increasing age among White, Black, Hispanic, Asian/Pacific Islander, and Native American inpatients. Mean HFRS was highest among Native American inpatients for the youngest age groups < 45 years, 45–49 years, 50–54 years, and 55–59 years ranging from 4.82 (SD = 4.39) to 6.65 (SD = 4.76). Mean scores were highest among Black inpatients for age groups 60–64 years, 65–69 years, 70–74 years, 75–79 years, 80–84 years, and 85–89 years ranging from 7.00 (SD = 5.39) to 9.94 (SD = 6.25). Alternatively, among the older age groups (65y +), White inpatients had the lowest mean HFRS compared to the minority races (with the exception of Native American inpatients in the 85–89 years and 90y + age groups). Among the oldest inpatients (90y +), the highest mean HFRS was observed among Asian/Pacific Islander (10.48, SD = 6.20) and Black inpatients (10.47, SD = 6.22). The percentile values showed a similar trend with median scores highest among younger Native American inpatients, while among the older inpatients median scores were highest among Black and Asian/Pacific Islander inpatients.

## DISCUSSION

We performed a nationwide assessment of frailty risk among US adult inpatients using the Hospital Frailty Risk Score (HFRS), a valid and accurate tool for identifying frailty risk among hospitalized patients.^24^ The initial validation of HFRS provided a description of low, intermediate, and high risk for frailty based on diagnoses and provides context for risk of post-admission outcomes (30-day mortality, longer length of stay, and re-admission) based on frailty risk category.^24^ Our study builds on these findings by providing normative values for HFRS in the US population stratified by age, sex, and race, which can allow assessment of inpatients for risk of frailty and associated outcomes using these norms. To the best of our knowledge, only one other study has provided normative values for a frailty index in a population-based sample, likely due to the difficulty in administering frailty assessments to a large sample of individuals.^31^ Normative values for the HFRS, an index based on more readily available ICD-10 codes, can help promote widespread use of frailty assessment in the hospital setting and comparison among inpatient populations.

Assessing inpatients for frailty risk can be beneficial in identifying patients at risk of adverse outcomes such as hospital-associated disability, increased length of hospitalization, and death.^10,16,32^ As the older adult population grows in size, this will be particularly important because older age is associated with higher frailty risk.^33,34^ In agreement with a recent systematic review and meta-analysis showing that frailty prevalence among community-dwelling adults increased from 11% among those aged 50–59 years to 51% among those aged 90y + ,^33^ our study identified that mean HFRS among US inpatients increases incrementally with increasing age, peaking among those in the oldest age group (90y +). The increase in frailty risk with age is likely due to multiple factors including physiologic decline, psychosocial changes, and multimorbidity.^34,35^ As life expectancy improves, it is possible that the prevalence of frailty and total time spent frail will increase among older adults^36^ making it an even more significant predictor of health outcomes during hospitalization.

We also present findings herein showing that older inpatients (65y +) that are female (compared to male) and racial minority (particularly Black, Hispanic and Asian/Pacific Islander compared to White) have higher mean and median HFRS. These findings are in line with other research among community-dwelling older adults that identified higher prevalence of frailty among women and Black and Hispanic older adults.^33,34,37,38^ Despite females living longer and representing an increasing proportion of hospitalizations among older age groups in the present study (see Table 2); the biological changes that occur with menopause in conjunction with psychosocial and behavioral changes with age likely contribute to increased frailty risk among older women.^39^ Additionally, while not directly measured as part of the HFRS, greater frailty risk among racial minority older inpatients might be associated with socioeconomic disparities and barriers to accessing affordable healthcare.^40,41^ Differences in environmental factors could also play a role in this observation as well.^42^

National norms for HFRS can be useful for both healthcare professionals and researchers. Clinically, the HFRS norms provide a standardized tool for assessing frailty risk among patients hospitalized in the USA based on age, sex, and race to identify those in need of additional supportive services to mitigate negative outcomes. For example, if a 70-year-old Black male patient was admitted with a HFRS of 9.0, this score could be compared to the national norms presented herein indicating that this patient’s HFRS is higher than the typical mean score for someone of a similar age, sex, and race and between the 50th and 75th percentile. A score of 9.0 puts this patient in an intermediate risk of frailty and poor outcomes post-admission indicating this patient might benefit from additional supportive services.^24^ In research, the norms can serve as a reference tool to compare study patients to a national average for hospital frailty risk to determine representativeness of research samples to the general inpatient population. The norms can also provide a method for comparative studies assessing frailty risk across geography, hospitals or hospital systems, and patient populations.

A major strength of this study is the use a national database that provides a representative sample of adult inpatients across the USA. The sample is racially diverse and spans ages from 18 to 90y + , though it does not provide data on sex outside of male and female and does not differentiate between sex assigned at birth and gender identity. Other limitations of the NIS database include not being able to distinguish between preexisting conditions and conditions at hospital admission,^43^ missing data for demographics and other variables, and differences in diagnostic coding across hospitals. Further, due to data de-identification by the AHRQ, we cannot state that all observations were unique. It is possible that a single patient was readmitted, and each hospitalization was documented as a separate observation in the database. This generally leads to overestimation of the variability and decreased probability of statistical significance, but these effects should be minimal considering the sample size of our study. The large sample size of the NIS also means that small differences that were detected may be statistically but not clinically significant, particularly since a minimum clinically important difference (MCID) for the HFRS has not been established. Additionally, the data used in this analysis was collected prior to the COVID-19 pandemic and thus may not reflect change to inpatient demographics, health, or frailty during the pandemic. It is expected, though not proven, that these changes would become less in the post-COVID era. Additionally, the use of ICD codes for assessing frailty via the HFRS might not capture all relevant clinical details related to frailty status including those related to social determinants of health, as the HFRS incorporates Z codes for mobility and caregiver dependency but not socioeconomic and psychosocial factors. Furthermore, diagnostic codes during hospitalization may not be finalized until discharge, which might present a barrier to practical use of the HFRS during the hospitalization itself ICD-10 coded admission diagnoses may still be used for an initial estimate of frailty in such circumstances. The HFRS may also be utilized on a larger scale by hospital administrators to estimate the rates of frailty at their institutions and provide adequate level of services for their patient base, and to encourage more widespread investigations of frailty in epidemiological studies that collect EHR data but not frailty assessments.

The study presents the national normative values for HFRS among US adult inpatients using the 2018 NIS database. Mean and median scores based on age, sex, and race are provided. Ultimately, these national norms can assist in rapid assessment of frailty risk, which can affect clinical care of patients by providing an easy method to identify risk across inpatients, and research practice by providing a tool that enables comparison of frailty across inpatient populations to a national standard.

## Data Availability

The data used in this analysis came from the US Government Agency for Healthcare Research and Quality and is publicly available (https://hcup-us.ahrq.gov/nisoverview.jsp).
